# Progress of Syphilology and Dermatology

**Published:** 1874-07

**Authors:** James Nevins Hyde

**Affiliations:** Lecturer on Dermatology and Syphilis, Rush Medical College, Chicago


ARTICLE I.—*Progress of Syphilology and Dermatology.* By JAMES NEVINS HYDE, M.D., Lecturer on Dermatology and Syphilis, Rush Medical College, Chicago.

1. On the Affections of Syphilis which tend to a Fatal Termination. BROUARDEL. (*La Gazette des Hopitaux*, April 14, 1874.)

2. On the Non-infecting Cephalic Chancre. Prof. J. PROFETA, with note by P. DIDAY. (*Annales de Dermatol. et de Syphilig.*, T. s. No. 3.)

3. On Syphilitic Angina. BUCQUOY. (*La France Medicale*, April 1, 1874.)

4. On Palmar and Plantar Syphilides, with especial reference to their study in Hereditary Syphilis. (*Madier-Champvermeil*, Paris, 1874.)

5. On Parasitic Melanodermia. FABRE. (*Le Progres Medical*, February 4, 1874.

6. On Buboes of the Inguinal Region. AUSKITZ. (*Amer. Jour. of Syph. and Derm.*, April, 1874.)

1. All syphilitic patients are not equally liable to the visceral disorders which ordinarily produce death. Certain classes of these seem especially predisposed to such complications, while others appear to be exempt.

The graver forms of syphilis occur when the precedent chancre has been misunderstood or undiscovered, when the sore has been purposely concealed from the attending physician, and when the first evidences of constitutional disease have failed to receive due attention—in short, the absence of treatment is productive of relative gravity of the disease.

Primitive sores, and so-called primary syphilis, rarely terminate fatally. Phagedena, ordinarily complicating non-infecting sores, is also established in some instances of syphilitic chancre, but terminates favorably in a large majority of cases.

It has been concluded that chancres contracted without coitus (vaginal touch of the practitioner, uncleansed instruments, etc.) require a more serious prognosis than others. But the determining causes in such instances are not different from those which obtain elsewhere. They are: misconstrued or neglected primary sore, and tardy treatment. Localized epidemics of syphilis, terrible in their gravity, illustrate this point. They originate in vaccinal syphilis, whose first phenomena are misunderstood and untreated.

In so-called secondary syphilis, M. Dubuc describes "malignant precocious syphilides." They become rapidly papular (rather than roseolous), and develop an embryonic cellular tissue, which, instead of becoming organized, rapidly degenerates. From this metamorphosis, of what Lancereaux terms "still born" tissue, ulceration, with deferred cicatrization, results. These cases are rapidly followed by grave complications and the development of embryonic tissue in the viscera. Iritis, single and double, is then frequent, and, what is worthy of note, the severe pain and photophobia of other patients is here wanting.

Precocious malignant syphilides are developed in the aged, the scrofulous, and those enfeebled by diathetic disorders. It was Ricord, who, observing the desperate tenacity, singular career and great gravity of one class of these complications, declared that there was a "scrofulate of pox."

Maladies complicating syphilis, are subject to a reciprocal influence, and are characterized by an irregular career, and inaptitude for relief, while they require indefinite treatment. Disorders of the lung, for example, in syphilitic patients, simulate tuberculosis. Gobler has relieved a certain proportion of these grave disorders with specific treatment; but it is impossible to generalize where lesions are so little understood, and so undistinguishable in the cadaver from those of non-specific disorders.

It is well known that the third period of syphilis is that of the greatest fatality. The characteristic of this period is the production of the gummata—neoplasms of embryonic tissue—which, unlike tubercle, are vascular, and therefore of superior vitality. Gummata belong to the cellular tissue, and determine in their vicinity diffuse inflammation and lesions of neighboring organs. When located in the subcutaneous cellular tissue, ulceration extends to the surface, and pus escapes, or a liquid resembling a solution of gum, which has given origin to the name. But the subcutaneous and sub-periosteal gummata do not endanger life—it is they which are located in organs essential to life—tertiary syphilides of the brain, respiratory passages, meninges, medulla, liver and cardiac muscle—which need to be considered. The groups which are of special importance, are those of the brain, respiratory passages and nervous centres.

(A.) *Lesions of the Central Nervous System.*—This division may be made to include disorders of the cranial and pericranial tissues, the meninges, the encephalic vessels, and the brain and spinal marrow. Periostoses of the cranial vault, rare upon the external table, are frequent on the internal face of the skull. The seat of predilection is the vicinity of the sella turcica, the basilar apophysis, and the base of the brain generally. Gummy tumors frequently result from these neoplasms—called by Virchow "gummy periostites." They contain the "dead bone" product of Lancereaux, and are prompt to disappear and leave depressions in the bony substance. Complete osseous perforation is more readily effected if the primitive lesion be located in the dura-mater.

Pachymeningitis occurs spontaneously with, or consecutive to, gummata. Virchow describes the diffuse gummata of the arachnoid as "gummy pachymeningites." Obliteration of the carotid at the level of the sylvian artery, and of the basilar trunk, has occurred from compression induced by an inflamed arachnoid. Virchow has also noted thrombosis, with obliteration of one lateral sinus. These disorders may produce all the symptoms of general paralysis.

As regards the vessels, syphilitic endarteritis and consecutive obliteration may occur. (Broadbent notes one fatal case.) Generally, however, the etiological conditions of endarteritis obtain in these cases—age and alcoholism.

The encephalon exhibits gummy tumors of poorly defined limits and diffuse periphery—isolated and self-limited when ancient or in full retrogression. These are genuine gummy inflammations, developing at the expense of the brain substance, and lie upon the convolutions, the crura, or the pons varolii. (Herard narrates cases of central lesion). Potain, Wagner, MacMowel, Charcot and Gombauld describe similar phenomena in the envelopes and substance of the spinal medulla.

Violent nocturnal cephalalgia, disappearing by day, and inducing insomnia, vertigo and vomiting, is common to all syphilitic periods, but the tertiary forms are obstinate, and persist through the day. The vomiting resembles that of meningitis; it is regurgitant and not accompanied by nausea. Exostoses and periostoses frequently co-exist. The iodide of potassium is not alone sufficient for a cure, but recourse must be had to mercury.

Most paralyses of the oculo-motorius are notably *not* dependent upon osteal or periosteal lesion, as they are consecutive to secondary accidents. Deafness may result from mucous patches of the eustachian tube. (Ricord.) Sudden and incomplete hemiplegia may result from a true apoplexy—inflammation having been developed from a gummy periostitis. Vascular obliteration from clot or circumferential meningitis, may produce slow or rapid softening from loss of blood supply.

Epileptiform seizures are less complete and more localized when syphilitic, than when uncomplicated. There is, in general, no *aura* nor alternate pallor and suffusion of the countenance.

(B.) *The Liver.*—Interstitial hepatitis and gummy tumors of the liver strangle the active agents of nutrition, and produce atrophy and amyloid degeneration of the entire gland. Vascular obliteration, here, begets ascites, and obliteration of the biliary ducts, icterus. In the examination of suspected cases, the volume of the liver will be found to vary within large limits. Projections, depressions, and lobar division, are frequent. (Riegel cites a case where one lobe seemed to float like a tumor in the right iliac region.) When the liver extends beyond the false ribs, it is difficult to cause the peritoneum to glide smoothly over its surface.

Syphilitic ascites is variable—reappearing and disappearing. It is, therefore, more curable in extreme cases than other forms. Icterus presents the same feature, and may produce almost a black discoloration of surface. Hæmorrhage from the stomach, bowels and nose may co-exist, and induce diagnostic errors.

(C.) Laryngeal œdema may originate in mucous patches of the vocal cords or adjacent membrane. (Cusco remarks that papulo-squamous syphilides select the larynx by preference.) Then may result, ulcers invading the trachea, aphonia from cord destruction or ulceration, and stenosis. (For an excellent review of the subject of syphilitic laryngeal stenosis, see article of Prof. Elsberg, an abstract of which appeared in the Progress, of this department of the JOURNAL, for May.)

2. Ricord, Cullerier, Langlebert and Galligo state that they have never observed non-infecting chancres in the cephalic region.

Others have admitted the possibility of their artificial production, but equally denied the fact of their clinical existence. To the experimental inoculation of Bossereau, Diday, Bennet, Rollet, Robert, Puche, and Buzenet, and the clinical observations of Devergie, Clerc, Fournier and others, Profeta adds three cases of interest.

In the first, that of an Italian musician, there existed on the right commissure of the lips, and the cheek and forehead of the same side, a vast chancre, produced by inoculation from a similar sore upon the right index, of irregular form, engorged but non-indurated base, overspread with crests and depressions, and covered with a yellowish, pultaceous, organic detritus. Its borders were well defined, but concealed fistulous tracts. In brief, it was a huge, serpiginous, perforating, phagedenic chancre. There was no trace of constitutional syphilis nor of adenopathy. (Ricord denominates the engorgement of the lymphatic ganglia the "syphilitic pulse.") A vesico-papular eruption, covered with crusts, extended over the trunk and limbs, evidently caused solely by the *acarus scabiei*, whose presence was detected. Profeta *inoculated himself* with the pus of this cephalic ulcer, and produced a soft chancre on his person, which was recognized by Colomioti, Meredyth, of London; Thiry, of Brussells; Kuss, of Strasbourg, and Pellizari, of Florence.*

The second case is that of a barber, who opened with his razor a suppurating inguinal bubo, symptomatic of multiple soft chancres, wounded his finger, and sucked the digital wound, which was covered with pus from the groin. He thereby inoculated a scratch upon the middle of his upper lip, and there resulted a large painful chancre of the index, secreting a sanguinolent pus, having clean cut edges, a greyish floor, and a reddish areola. Axillary adenopathy of inflammatory type followed. Both the upper and under

* When it is remembered that Profeta was a pupil of Pellizari, his courageous self-inoculation can readily be understood. He and his master are both ardent advocates of the dualistic doctrines on syphilis, and, as can be seen, they give practical evidence of their convictions. It was another pupil of Pellizari, viz., Bargioni, who first demonstrated the inoculability and infectiousness of syphilitic blood in his own person. That experiment is considered decisive by all authorities, since Bargioni, in accordance with his expectations and in consequence of his inoculation, suffered from constitutional disease.

lips soon presented similar sores, without, however, lymphatic engorgement in the neighborhood.

The third patient had a very extensive non-infecting chancre of the penis, with gangrenous complication. There was also a consecutive sore upon the scrotum, and the right ala of the nose was entirely covered with a clean-cut, non-infecting sore, of a livid areola and a gangrenous aspect. The patient had had a pruriginous affection of the nostril, which he frequently scratched, and complete inattention to cleanliness had resulted in infection. The nasal sore was soon surrounded with numerous smaller and similar lesions, which rapidly united, to form an enormous phagedenic facial chancre.

Diday appends the following conclusion:

It is clear that the chancrous virus (the pus of a soft chancre) can be inoculated upon the face, just as in other regions. But is this easy of accomplishment? If the relatively small number of cephalic chancres be considered, in connection with the fact that the application of a contagious pus upon a sub-epidemic surface has been in each case necessary, it may be concluded that contagion, in order to produce a facial ulcer, requires something more than is requisite for a similar effect upon the genitals. All the facial sores described above were of phagedenic type. Would it be exceeding the limits of legitimate deduction to conclude from this circumstance, that there existed in each case an exceptional predisposition to the ulcerative process, and that on this condition only was the contagion of the chancroid ulcer possible of production upon the face?

3. A shoemaker, thirty years of age, of robust constitution and with every appearance of good health, was admitted to the Hopital Cochin, Feb. 23, 1874, for sore throat, from which he had suffered for five or six weeks. Examination disclosed on the left side an extensive irregular ulceration, with clean-cut edges, involving the superior portion of the anterior pillar of the fauces, the adjacent tonsil and the left border of the velum and uvula. This vast ulcer had a grayish base, a periphery of brilliant red color, and a swollen, angry-looking areola. The uvula, increased to twice its normal size, rested inactive upon the base of the tongue. Similar lesions were noted deeper in the pharynx upon its posterior wall, which was, to a great extent, covered with irregular deep excavations, smeared with a grayish and adherent secretion. The laryngoscope brought to light every feature of these enormous ulcers, which extended upward and downward as far as the eye could penetrate. Below, they appeared to be limited by the rim of the larynx. The latter was somewhat reddened, but the epiglottis and vocal cords exhibited no alteration. The nasal fossæ presented lesions of a color similar to that seen in the larynx. There was no maxillary adenopathy. The voice was unaltered, the odor of the breath occasionally fetid. Pain existed only in deglutition. There was some ear-ache of the left side, but no deafness.

Antecedents: At eighteen years of age an indurated chancre appeared upon the prepuce; treated in hospital for one month. Two years afterward he was treated in hospital for left iritis during another month. Inequality of pupil movement remained. Somewhat later, lesions of the throat appeared, similar to those of the present date. Eighteen months afterward, a pustule on the left shoulder left a large white stellate cicatrix; a smaller cicatrix was found upon the left leg. Ecthyma, and possibly rupia, had evidently existed. Fifteen months before, an abscess had burst into the buccal cavity, near the upper canine tooth.

This latter affection had begun with an insignificant ulceration, which had broadened and deepened, and in less than one month attained its present dimensions. The patient experienced frequent nocturnal headaches, but had no periostitis. No regular course of treatment had been pursued. There was room for some suspicion of the coexistence of scrofula. The patient had had sore eyes since he was four years old, but never suffered during infancy from ganglionic enlargement.

There are but two chronic disorders which are capable of producing these lesions. They are scrofula and syphilis. The signs of the latter are distinct in the case related, though scrofulous antecedents are not wanting.

Primitive, secondary and tertiary lesions of the throat are all definite and different. Chancres from *coitus ab ore*, and unclean instruments, (aurists', especially,) are indolent, superficial, painful, indurated, and accompanied by non-suppurative adenopathy.

The tonsils, says Fournier, are veritable nests for syphilides. Here also roseola and erythema are discoverable, allied to their congeners upon the skin. Mucous patches, however, preceded by slight erosion, are the commoner lesions. They are small, flattened, slightly granulating elevations, ashy-gray or opaline in color, sometimes extensive and sometimes involving the salmucous derma.

Tertiary syphilitic throat affections are always ulcerative, either *ab origine* or consecutive to gummata. Of the former class are the deep red ulcers upon the tonsils, velum and pharynx, having a damask colored and swollen areola, a grayish base, and sharply defined borders. Painful or not, they interfere with deglutition, and produce a guttural voice. The odor of the breath is fetid, a puriform mucus is expectorated. They may perforate periosteum and bone, injure the vascular tissues to an extent requiring ligature of the carotid, involve the eustachian tube, and produce excruciating ear-ache, or penetrate to the larynx and finally necessitate tracheotomy. Of the latter class are the little tumors of the velum, at first hardly sensible, which eventuate in redness, swelling, softening and disintegration of the gumma in an icherous pus. The resulting ulcers do not differ from those described above. In the case whose details are cited, there was evidently a serpiginous tertiary ulcer, whose gummy origin could not be positively affirmed.

Malignant scrofulous angina equally produces profound and destructive ulceration. These lesions, however, display irregular and sinuous edges, their floors are indolent and memelonnated. A dirty, yellowish tissue is found beneath the muco-purulent secretion which covers them. They originate in follicular enlargements, colored like lees of urine, (Paul) or appear like white prominences (Bozin, Lailler, Isambert). According to some they respect the tonsils and larynx. Relatively rare, they are, however, often distinguished with great difficulty from the lesions of syphilis.

4. The limits of this article forbid the presentation of extended extracts from this admirable monograph. We have space merely for the briefest reference to the ideas of the author. He combats the generally received opinion that pemphigus and squamous syphilides are the sole manifestations of syphilis upon the palmar and plantar regions.

These lesions, he advances, should be considered merely as accessory elements—epiphenomena—complicating and masking other pathological conditions. These other pathological conditions are those found elsewhere upon the integument of syphilitic patients. In the case of an adult who does not labor with the hands nor make excessive use of the feet, the palmar and plantar epidermis is thicker than in any other region of the body. Now the degree of this thickness bears a constant ratio to the repetition and intensity of pressure and friction upon these surfaces. If to this be added the pathological irritation of the syphilitic virus, there is excessive production of epidermis; and squamæ result. In the case of an artisan with callous hands, the latter element will more surely predominate and conceal from observation the deep lesions of the integument.

If the hand of an infant affected with hereditary syphilis be attentively examined, it will be found entirely virgin in respect to normal epidermic accumulations.* Here, however, the slightest shade of erythema, the most diverse forms of papules can be distinguished, precisely as upon other surfaces where there is no tendency to epithelial accumulation.

The squamæ, therefore, are to be regarded as complications; and the classification of the syphilides of the palm and sole should not differ, except in minor points, from that of the other phenomena of the disease.

This is the key note of the treatise. We append, in conclusion, the author's novel classification of general syphilides:

* The relative thickness of the epidermis in the unused hand of the infant and the adult, has not yet, so far as we know, been accurately determined. It is probable that the apparent excess of epidermal tissue in the palmar and plantar regions which have not been subjected to friction, is occasioned by the elevation of this component of the cutis by the dermal papillæ. In these localities the latter are not only large but numerous, and if a very dense epidermis were superimposed, the inequalities of the surface would be eradicated. Every one knows that this is not the case, but, like the "hills and valleys" of Rindfleisch, the surface is seamed with innumerable ridges.

The exquisite beauty of the rosy-pink tint in an infant's palm, is undoubtedly due to the approach of the vascular currents to the surface, in consequence of the delicacy of the epithelium. It is a peculiarity which rapidly disappears when the hand is once brought into use. Wilson estimated the epithelium of the palm of an adult hand, not exposed to manual labor, to be one-fourth of a line in thickness.

I.

*Hyperæmic Syphilides.*

| | | |
|---|---|---|
| Circumscribed: | Roseola of adults—acquired syphilis. | |
| Diffuse: | Erythema of adults. | |
| | Erythema of infants—hereditary syphilis. | |

II.

*Neoplastic Syphilides.*

| | | | *Complications* | |
|---|---|---|---|---|
| *a.* Simple: | Circumscribed. | Papule. | Squamous. | |
| | | Tubercle. | Horny. | |
| | Diffuse. | Psoriasis of adults. | Humid. | Ulcero-squamos. |
| | | Erythema squamosum of infants. | Ulcerative. | Rupia. |
| *b.* Pustular: | Seniform. | Horny. | | |
| | Pitted. | Squamous. | | |
| | Ecthymatous. | | | |
| *c.* Vesico-Bulbous: | Syphilitic Herpes. | | | |
| | Bulbous Erythema of infants. | | | |
| | Pemphigus. | | | |
| *d.* Ulcerative: | Superficial. | Ulcerative Erythema. | | |
| | Deep. | Gummata. | | |

5. It is difficult not to recognize a connection of causality between phthisiasis and melanodermicæ in the seven cases reported by Fabre. There is, in these disorders, a sepia-brown discoloration of the epidermis, much more intense where the parts are concealed by clothing and protected from friction. The head, hands, feet and neck are most often exempt, or less darkly tinted. The fœdo of the articulations are not generally more discolored than other parts, and the mucous membranes are generally unaffected. While the skin seems dry and rugous, the perspiratory function is illy performed. The affection attacks those in good health, as as well as those enfeebled by age, privation, excess or other disorders.

The author endeavors to maintain that the so-called cachectic melanodermia of Boucher, Gillet and Ponchet, is really of parasitic origin. Certainly, if the cachexia be the *predisposing* cause of this malady, it would seem to be clear that the parasite is the *exciting* cause. This once removed, the pigmentation commences to disappear. The Germans have called the affection "Pseudo bronze-disease," and the "Vagrant's malady." M. Ball (Dict. Encyclop., T. xi) declares that there is danger of a mistaken diagnosis, in view of the profound physical depression which usually coexists, and which, in many points, resembles the asthenia produced by Addison's disease.

6. The name of Heinrich Auspitz is sufficient to attract attention to this paper, even were it not as it is, an exhaustive discussion of the subject of inguinal buboes. The anatomy of the inguinal and femoral ganglia is described in a most thorough analysis of the results of numerous dissections, and illustrated with plates which are novel to the surgeon and the anatomist of our country. The article in question, is the first of ten lectures delivered at the Allgemeinen Paliklinik in Vienna, last October, and is translated from the *Archiv. fur Dermatologie und Syphilis*, of 1873.

We remark of the translation; it is so miserably executed, that it is at times almost impossible to seize the meaning of the author. Grammatical errors are the least of the faults evident. Long and complicated sentences invite the reader to enter a maze, from which he extricates himself only with the greatest difficulty. Idiomatic expressions are everywhere to be found, and the article is almost valueless to the average student in consequence of these defects.

This would be hardly deserving of mention, were it not for the fact that the Journal which produces Auspitz's lscture in this half English dress, is one of the very best medical periodicals in this country. The literary and scientific standard of the management is infinitely superior to that of the majority of our medical publications; and we look to it for better things. We desire that it shall speak *ex-cathedra* to the profession in this country and abroad, and are confident that the criticism we offer will be received in the kindly spirit in which it is tendered.

